# Body water conservation through selective brain cooling by the carotid rete: a physiological feature for surviving climate change?

**DOI:** 10.1093/conphys/cow078

**Published:** 2017-02-14

**Authors:** W. Maartin Strauss, Robyn S. Hetem, Duncan Mitchell, Shane K. Maloney, Haley D. O'Brien, Leith C. R. Meyer, Andrea Fuller

**Affiliations:** 1 Brain Function Research Group, School of Physiology, Faculty of Heath Sciences, University of the Witwatersrand, Johannesburg, 2193, South Africa; 2 Department of Environmental Science, University of South Africa, Johannesburg, 1709, South Africa; 3 School of Animal, Plant and Environmental Sciences, Faculty of Science, University of the Witwatersrand, Johannesburg, 2050, South Africa; 4 School of Anatomy, Physiology, and Human Biology, University of Western Australia, Perth, WA 6009, Australia; 5 Department of Anatomy and Cell Biology, Oklahoma State University Center for Health Sciences, Oklahoma, OK 74107, USA; 6 Department of Paraclinical Sciences, Faculty of Veterinary Science, University of Pretoria, Pretoria, 0110, South Africa

**Keywords:** Artiodactyl success, brain temperature, carotid arterial blood temperature, osmoregulation, physiological plasticity, rostral epidural rete mirabile

## Abstract

Some mammals have the ability to lower their hypothalamic temperature below that of carotid arterial blood temperature, a process termed selective brain cooling. Although the requisite anatomical structure that facilitates this physiological process, the carotid rete, is present in members of the Cetartiodactyla, Felidae and Canidae, the carotid rete is particularly well developed in the artiodactyls, e.g. antelopes, cattle, sheep and goats. First described in the domestic cat, the seemingly obvious function initially attributed to selective brain cooling was that of protecting the brain from thermal damage. However, hyperthermia is not a prerequisite for selective brain cooling, and selective brain cooling can be exhibited at all times of the day, even when carotid arterial blood temperature is relatively low. More recently, it has been shown that selective brain cooling functions primarily as a water-conservation mechanism, allowing artiodactyls to save more than half of their daily water requirements. Here, we argue that the evolutionary success of the artiodactyls may, in part, be attributed to the evolution of the carotid rete and the resulting ability to conserve body water during past environmental conditions, and we suggest that this group of mammals may therefore have a selective advantage in the hotter and drier conditions associated with current anthropogenic climate change. A better understanding of how selective brain cooling provides physiological plasticity to mammals in changing environments will improve our ability to predict their responses and to implement appropriate conservation measures.

## Teaser

Mammals with a carotid rete are capable of reducing brain temperature below that of carotid blood temperature, termed selective brain cooling. In artiodactyls, selective brain cooling conserves body water and may provide them with a selective advantage in conditions of increased temperature and aridity.

## Introduction

**Figure 1:**
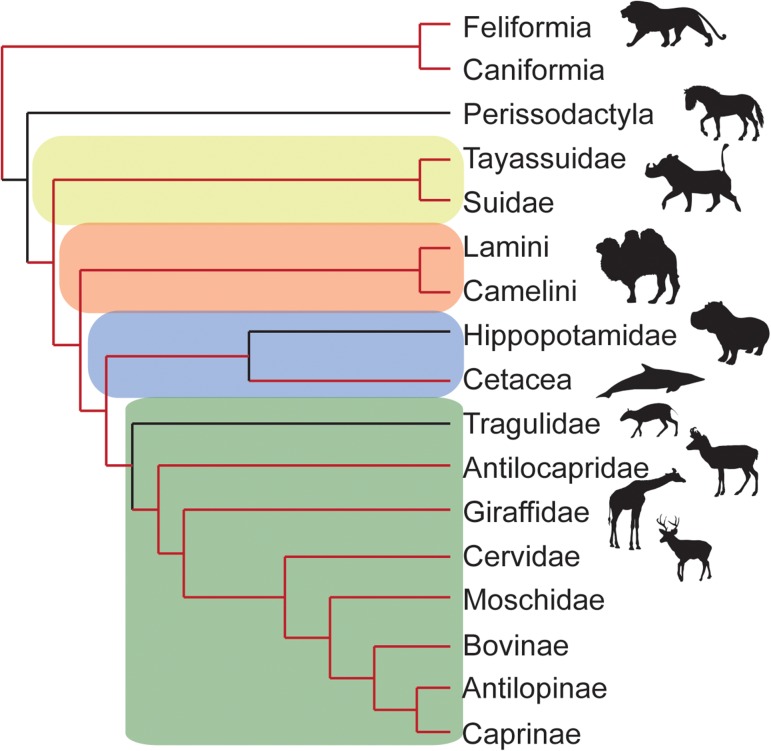
Phylogenetic tree indicating the relationship between the Cetartiodactyla, Perissodactyla and the Carnivora, represented by the cats and dogs (adapted from Hassanin *et al*., 2012). Red branches indicate clades with a carotid rete, capable of selective brain cooling. Black branches designate the absence of a carotid rete (Hippopotamidae largely data deficient). Also depicted in various shades are the Ruminantiamorpha (green), Whippomorpha (blue), Camelidamorpha (orange) and Suinamorpha (yellow).

The carotid rete, or rostral epidural rete mirabile, is an intracranial vascular structure, near-ubiquitous and often elaborate in the Ruminantiamorpha, Whippomorpha, Camelidamorpha and Suinamorpha, collectively known as the Cetartiodactyla (Fig. 1; nomenclature *sensu* Spaulding *et al*. (2009)). The carotid rete is present also, often in a rudimentary or primitive form, in a number of laurasiatherian mammals (Ask-Upmark, 1935; du Boulay and Verity, 1973), including cats (Kamijyo and Garcia, 1975), in which it is extracranial (Daniel *et al*., 1953), and domestic dogs (*Canis lupus familiaris*; Daniel *et al*., 1953; Gillilan, 1976). Primates, many small-mass mammals (for example, rodents, lagomorphs and ‘insectivores’) and perissodactyls (horses, tapirs and rhinoceroses), a sister group of the artiodactyls (Hassanin *et al*., 2012), have no carotid rete (Ask-Upmark, 1935; Sisson and Grossman, 1967; Gillilan, 1974). In artiodactyls (e.g. antelopes, cattle, sheep and goats), anatomical investigations (Ask-Upmark, 1935; Daniel *et al*., 1953; Gillilan, 1974; Carlton and McKean, 1977; Frąckowiak *et al*., 2015; Kiełtyka-Kurc *et al*., 2015), including the identification of osteological correlates in extant and extinct artiodactyls (O'Brien, 2016), and physiological studies (Johnsen *et al*., 1987; Mitchell *et al*., 1997; Fuller *et al*., 1999b; Maloney *et al*., 2002; Lust *et al*., 2007; Hebert *et al*., 2008; Hetem *et al*., 2012; Strauss *et al*., 2016) have confirmed the presence of the rete and its functionality in virtually all of the extant terrestrial artiodactyls, with informative exceptions (Fig. 1). In the vast majority of terrestrial artiodactyls, the carotid rete is found in lieu of the internal carotid artery and serves as the main supply of oxygenated blood to the brain (Schummer *et al*., 1981; Wible, 1984; Frąckowiak, 2006; O'Leary, 2010; O'Brien, 2015). An exception is the Tragulidae, which consist of three genera of small, forest-dwelling antelope that have retained an internal carotid artery instead of a carotid rete (Fukuta *et al*., 2007; O'Brien, 2015). Whether their unique cranial vasculature is a plesiomorphic (*sensu* Janis, 1984) or apomorphic characteristic (e.g. Clauss and Rössner, 2014) has not been resolved. The Hippopotamidae also are artiodactyls, and a historic paper identifies hippopotamuses as having a carotid rete (Chapman, 1881), but it is indistinct (du Boulay and Verity, 1973; O'Brien, 2016). The status of the rete in Hippopotamidae requires further investigation using modern techniques, because it was identified before the anatomical techniques of resin injection and maceration, for exploring vascular systems, became available.

The function of the carotid rete in the Cetacea is unknown, but in the terrestrial artiodactyls, cats and dogs, it is a heat exchanger that can be used to cool the brain, but importantly, the hypothalamic region, below the temperature of carotid arterial blood. This phenomenon, known as ‘selective brain cooling’, first was described in a domestic cat (*Felis catus*) almost 50 years ago (Baker and Hayward, 1967). The brain, as a metabolically active organ, usually has a temperature higher than that of the arterial blood perfusing it. The heat produced by the brain is removed by that blood, meaning that in the absence of a carotid rete, the brain is ~0.5°C warmer than arterial blood leaving the heart (Fuller *et al*., 2000; Maloney *et al*., 2009). The carotid rete, carrying arterial blood destined for the brain, is surrounded by venous blood in either a cavernous sinus (artiodactyls) or a pterygoid sinus (felids; Daniel *et al*., 1953). The venous blood is derived from the maxilloturbinates and other mucous surfaces of the mouth and nose, where it is cooled to well below arterial blood temperature by evaporation of water into inspired air, whether or not the mammal is panting (Kuhnen and Jessen, 1991). The thin walls and large surface area of the carotid rete vessels allow for efficient heat exchange between the arterial and venous blood, the result being that the arterial blood exiting the carotid rete into the brain and, subsequently, the hypothalamic tissue in that region, can be more than 1°C cooler than arterial blood entering the rete (Maloney *et al*., 2007).

Soon after the heat-exchange function of the carotid rete was discovered, investigations of selective brain cooling in domesticated and habituated wild mammals in captivity led to the conclusion that selective brain cooling served to protect the brain from reaching dangerously high temperatures (Baker and Hayward, 1967, 1968; Magilton and Swift, 1968; Baker, 1972, 1979; Taylor and Lyman, 1972; Baker and Chapman, 1977; Mitchell *et al*., 1987). That conclusion was influenced heavily by one measurement of selective brain cooling. Taylor and Lyman (1972) reported that brain temperature after induced exercise in habituated Thomson's gazelle (*Gazella thomsonii*) was as much as 2.7°C lower than carotid arterial blood temperature, a magnitude of selective brain cooling never seen before or since by anyone else, in any mammal. A protectionist function fitted well with the perceived vulnerability of brain tissue to thermal damage *in vitro* (Burger and Fuhrman, 1964). Only when it was shown that goats (*Capra hircus*) could withstand brain temperatures of 42.5°C for an hour without any apparent ill effect (Caputa *et al*., 1986b) did it become apparent that the brain was not as vulnerable to thermal damage as believed previously (Mitchell *et al*., 2002). Indeed, rather than the brain, it is the tissue of the gastrointestinal tract that is most susceptible to thermal damage (Braasch, 1964), attributable to reduced splanchnic blood flow and endotoxin leakage (Leon and Helwig, 2010). Subsequent studies of free-living and unrestrained mammals have revealed that selective brain cooling is not obligatory at high body temperatures (Mitchell *et al*., 2002) and, as first noted in a laboratory study on goats, it is part of the normothermic thermoregulatory repertoire of artiodactyls (Kuhnen and Jessen, 1991). Rather than functioning primarily to protect the brain from thermal damage, selective brain cooling modulates the use of body water for thermoregulation (Jessen *et al*., 1998). By reducing the temperature of the hypothalamus, where the temperature sensors that provide the internal drive for heat loss are located, selective brain cooling reduces evaporative water loss (Kuhnen, 1997; Strauss *et al*., 2015). In the context of this alternative role for selective brain cooling, now described in textbooks of animal physiology (for example, Willmer *et al*., 2009; Withers *et al*., 2016), we provide here a perspective on the evolutionary and functional significance of selective brain cooling and its potential to provide physiological plasticity for artiodactyls facing hotter and drier environments associated with current climate change.

## Factors controlling selective brain cooling

Control mechanisms govern the onset and degree of selective brain cooling in mammals with a carotid rete. The finding that selective brain cooling typically is exhibited by tame or habituated mammals when exposed to heat or exercise indicated that a primary input to the control of selective brain cooling is the mammal's internal temperature (Jessen *et al*., 1998). However, the absence of selective brain cooling in hyperthermic artiodactyls, particularly in free-living mammals during intense exercise, revealed that there also are non-thermal inputs in the control of selective brain cooling, and that these inputs can override thermal inputs (Mitchell *et al*., 2002; Fuller *et al*., 2014).

### Thermal inputs

**Figure 2:**
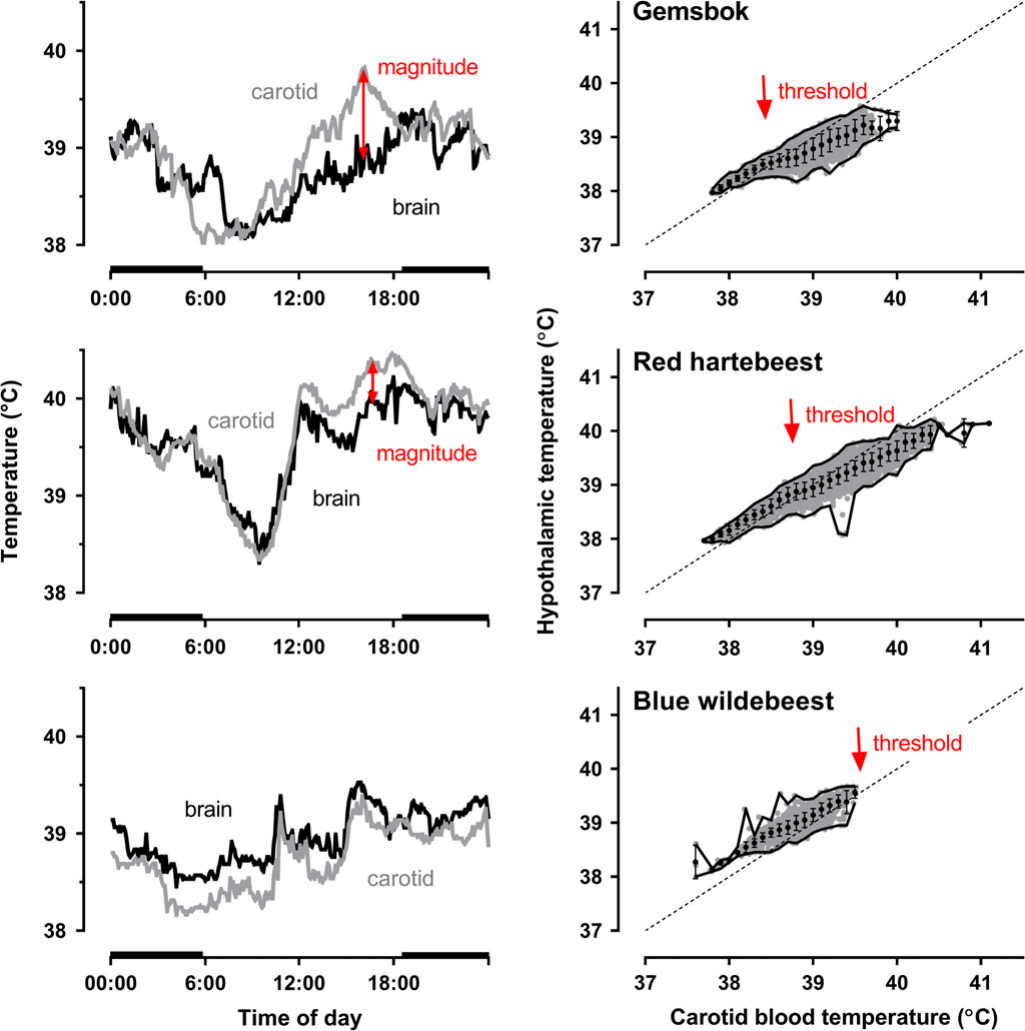
Left panels show the 24 h carotid blood and hypothalamic temperature profiles of a single gemsbok *Oryx gazella* (upper), red hartebeest *Alcelaphus buselaphus* (middle) and blue wildebeest *Connochaetes taurinus* (lower), for a single day, when the animals were free living in the same conditions in the Northern Cape Province, South Africa. Red arrows represent the magnitude of selective brain cooling within the 24 h period. Horizontal black bars indicate night time. Right panels show the correlation of hypothalamic temperature against carotid arterial blood temperature (grey circles) as well as hypothalamic temperature (mean ± SD) for every 0.1°C bin of simultaneous carotid arterial blood temperature, in the same gemsbok (upper), red hartebeest (middle) and blue wildebeest (lower) over a 5 day period during which they were exposed to the same environmental conditions. The boundary lines demonstrate the minimum and maximum hypothalamic temperatures in each bin. The diagonal line is the line of identity. Red arrows indicate the respective observed threshold temperatures for selective brain cooling; in the blue wildebeest the threshold was not reached within the range of measurement. Data from Strauss *et al*. (2016).

The typical relationship between hypothalamic temperature and pre-rete carotid arterial blood temperature in artiodactyls with carotid retes is shown in Fig. 2 (right panels). At the lower carotid arterial blood temperatures, brain temperature is higher than, and runs in parallel to, carotid arterial blood temperature, as illustrated for one gemsbok (*Oryx gazella*, upper panel), one red hartebeest (*Alcelaphus buselaphus*, middle panel) and one blue wildebeest (*Connochaetes taurinus*, lower panel) over the same 5 day period. In that temperature regime, in all mammals, whether or not they have a carotid rete, hypothalamic temperature generally exceeds carotid arterial blood temperature by about 0.2–0.5°C (Maloney *et al*., 2007). As carotid arterial blood temperature increases, hypothalamic temperature uncouples from carotid arterial blood temperature, as selective brain cooling ensues in those species with a carotid rete. Figure 2 also illustrates two variables that can be used to characterize selective brain cooling (red arrows): the temperature at which hypothalamic temperature and carotid arterial blood temperature are equal, which is the threshold temperature for selective brain cooling (Kuhnen and Jessen, 1991), and the extent to which brain temperature drops below carotid arterial blood temperature, the magnitude of selective brain cooling. The threshold temperature for selective brain cooling can differ, not only between species simultaneously exposed to the same environmental conditions (Fig. 2), but also both between individuals within a species (Strauss *et al*., 2016) and within an individual when exposed to different environmental conditions (Hetem *et al*., 2012), with the threshold temperature for selective brain cooling being reduced under high heat loads (Strauss *et al*., 2016).

The finding that hyperthermia is a not a prerequisite for selective brain cooling is confirmed by measurements of the threshold temperature in many species (Table 1); it typically lies between 38 and 39°C (Table 1), close to the modal and mean body core temperature of artiodactyls (Hetem *et al*., 2016). In captive mammals, the magnitude of selective brain cooling typically increases with increasing carotid arterial blood temperature, but in free-living wild mammals it is more variable. Figure 2 (left panels) illustrates the carotid arterial blood and hypothalamic temperatures of three individual artiodactyls, a gemsbok (upper panel), a red hartebeest (middle panel) and a blue wildebeest (lower panel), living free in the same habitat and measured over the same 24 h period (Strauss *et al*., 2016). Like other large mammals (Mitchell *et al*., 2002; Hetem *et al*., 2016), these antelope had a 24 h rhythm of arterial blood temperature, with a trough soon after dawn and a peak in the late afternoon (Fig. 2, grey line). Hypothalamic temperature (Fig. 2, black line), which is determined mainly by post-carotid rete arterial blood temperature (Hayward *et al*., 1966), had a 24 h pattern similar to that of carotid arterial blood temperature, and the individuals from the three species exhibited a pattern of selective brain cooling similar to that typically exhibited by other free-living artiodactyls (Jessen *et al*., 1994; Mitchell *et al*., 1997; Hetem *et al*., 2012; Strauss *et al*., 2016). As carotid arterial blood temperature approached its 24 h peak, hypothalamic temperature uncoupled from carotid arterial blood temperature, and selective brain cooling ensued. Although selective brain cooling could be implemented at any time of the day (e.g. Fig. 2, middle left panel), its magnitude typically was greatest (0.5–1.5°C; Table 1) around the time of the 24 h peak in carotid arterial blood temperature. At that time of day, environmental heat load was decreasing, and the antelope usually were involved in low-intensity activities, such as rumination and grazing. Despite strong thermal inputs at that time of day, however, selective brain cooling in artiodactyls can be modulated by non-thermal inputs, such that its magnitude can be further increased or it can even be completely abolished.

**Table 1:** The threshold temperature (mean ± SD, where originally reported) and maximum magnitude of selective brain cooling reported (or inferred) from studies of selective brain cooling in artiodactyls

Species (sample size, *n*)	Selective brain cooling		Notes	Reference
	Threshold (°C)	Magnitude (°C)		
Domestic or habituated animals studied in controlled laboratory conditions, unless otherwise indicated				
Goat *Capra hircus* (6)	Not reported	2.5	Heat exchanger	(Caputa *et al*., 1986a)
Goat *Capra hircus* (3)	38.8 ± 0.1	1.2	Heat exchanger	(Kuhnen and Jessen, 1991)
Goat *Capra hircus* (3)	39.1 ± 0.1	0.5	Heat exchanger and high humidity	(Kuhnen and Jessen, 1992)
Goat *Capra hircus* (3)	39.2 ± 0.1	1.2	Heat exchanger and low humidity	(Kuhnen and Jessen, 1992)
Goat *Capra hircus* (3)	38.9	1.5	Heat exchanger	(Kuhnen, 1997)
Goat *Capra hircus* (3)	39.0	0.3	Free-living, euhydration	(Jessen *et al*., 1998)
Goat *Capra hircus* (3)	38.9	0.8	Free-living, dehydration	(Jessen *et al*., 1998)
Goat *Capra hircus* (5)	39.3 ± 0.1	0.7	Hydrated and exercise	(Baker and Nijland, 1993)
Goat *Capra hircus* (5)	39.3	1.2	Dehydrated and exercise	(Baker and Nijland, 1993)
Ox *Bos taurus* (11)	39.1	0.8	Heat exposure	(Chesy *et al*., 1983)
Ox *Bos taurus* (3)	40.3	1.5	Exercise	(Chesy *et al*., 1985)
Sheep *Ovis aries* (3)	Not reported	0.6	Heat exchanger and heat exposure	(Maloney and Mitchell, 1997)
Sheep *Ovis aries* (4)	Not reported	1.0	Room temperature	(Laburn *et al*., 1988)
Sheep *Ovis aries* (4)	Not reported	1.0	Heat exposure	(Laburn *et al*., 1988)
Sheep *Ovis aries* (4)	Not reported	1.0	Febrile, induced	(Laburn *et al*., 1988)
Sheep *Ovis aries* (4)	Not reported	0.5	Exercise	(Laburn *et al*., 1988)
Sheep *Ovis aries* (4)	Not reported	0.8	Heat exposure	(Nijland *et al*., 1990)
Sheep *Ovis aries* (4)	Not reported	0.6	Cold exposure	(Nijland *et al*., 1990)
Sheep *Ovis aries* (4)	Not reported	0.9	Febrile, induced	(Nijland *et al*., 1990)
Sheep *Ovis aries* (9)	39.1	0.4	Water deprivation and heat exposure	(Fuller *et al*., 2007)
Sheep *Ovis aries* (9)	39.5 ± 0.5	1.5	Water deprivation and heat exposure	(Strauss *et al*., 2016)
Sheep *Ovis aries* (5)	39.1 ± 0.5	0.5		(Maloney *et al*., 2007)
Pig *Sus scrofa* (4)	38.9	0.9	Thermoneutral	(Fuller *et al*., 1999a)
Pig *Sus scrofa* (2)	Not reported	0.8	Heat stress	(Fuller *et al*., 1999a)
Pig *Sus scrofa* (1)	Not reported	0.3	Cold stress	(Fuller *et al*., 1999a)
Camel *Camelus dromedarius* (2)	38.0	1.0	At rest	(Schroter *et al*., 1989)
Camel *Camelus dromedarius* (1)	39.5	1.5	Exercise, hydrated and dehydrated	(Schroter *et al*., 1989)
Reindeer *Rangifer tarandus* (3)	38.7 ± 0.2	1.0	Heat exchanger	(Kuhnen and Mercer, 1993)
Reindeer *Rangifer tarandus* (3)	39.5 ± 0.3	0.5	Exercise	(Kuhnen and Mercer, 1993)
Thomson's gazelle *Gazella thomsonii* (5)	39.4	2.7	Exercise	(Taylor and Lyman, 1972)
Free-living wild animals with free access to normal behaviour				
Black wildebeest *Connochaetes gnu* (4)	38.9 ± 0.2	0.4		(Jessen *et al*., 1994)
Eland *Tragelaphus oryx* (1)	40.0	0.4		(Fuller *et al*., 1999b)
Gemsbok *Oryx gazella* (4)	39.8 ± 0.4	0.4		(Maloney *et al*., 2002)
Gemsbok *Oryx gazella* (4)	39.5 ± 0.9	0.9		(Strauss *et al*., 2016)
Kudu *Tragelaphus strepsiceros* (4)	39.3 ± 0.7	0.5	Febrile, naturally	(Hetem *et al*., 2008)
Kudu *Tragelaphus strepsiceros* (4)	38.8 ± 0.1	0.2	Afebrile	(Hetem *et al*., 2008)
Arabian oryx *Oryx leucoryx* (4)	37.8 ± 0.1	1.4		(Hetem *et al*., 2012)
Springbok *Antidorcas marsupialis* (2)	39.2 ± 0.2	0.5		(Mitchell *et al*., 1997)
Pronghorn *Antilocapra americana* (2)	39.5	0.5		(Lust *et al*., 2007)
Blue wildebeest *Connochaetes taurinus* (6)	39.3 ± 0.4	1.1		(Strauss *et al*., 2016)
Red hartebeest *Alcelaphus buselaphus* (5)	39.4 ± 0.6	1.0		(Strauss *et al*., 2016)

### Non-thermal inputs

#### Cranial sympathetic tone

**Figure 3:**
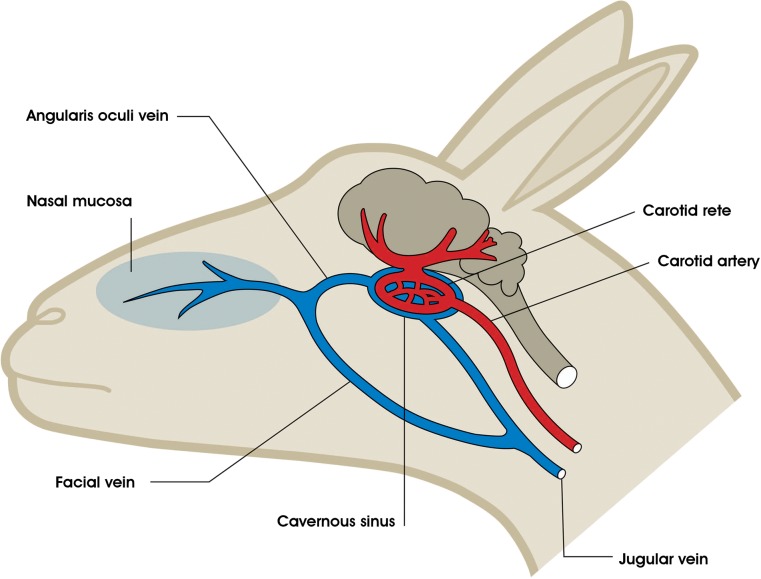
Diagram illustrating the position of the carotid rete in artiodactyls, located within a cavernous sinus at the base of the brain, as well as the main arterial blood supply to the brain, via the carotid rete. During selective brain cooling, cool venous blood from the nasal mucosa drains into the cavernous sinus via the angularis oculi vein (as well as some deeper veins; see Fuller *et al*., 2011). During high sympathetic activity, the cooled venous blood draining from the nasal mucosa largely bypasses the cavernous sinus as it is shunted via the facial vein, thereby attenuating selective brain cooling. Diagram adapted from Jessen (1998).

A role for sympathetic nervous system activity in the modulation of selective brain cooling was revealed through a series of elegant experiments on reindeer (*Rangifer tarandus*; Johnsen *et al*., 1985, 1987; Johnsen and Folkow, 1988). Experiments on habituated artiodactyls showed that selective brain cooling could be controlled by directing the passage of venous blood draining the evaporating surfaces of the head either to the cavernous sinus, classically via the angularis oculi veins, or to the jugular vein via the facial vein, bypassing the cavernous sinus (Fig. 3). The default direction of blood flow appears to be via the cavernous sinus, so increased cranial blood flow in heat-stressed artiodactyls (Maloney and Mitchell, 1997; Vesterdorf *et al*., 2011) led to increased flow of cooled venous blood to the cavernous sinus; hence, increased selective brain cooling. Increased cranial sympathetic nervous system activity, however, led to simultaneous contraction of a muscular sphincter in the angularis oculi vein (α-adrenergic) and dilatation of a similar sphincter in the facial vein (β-adrenergic), resulting in venous blood bypassing the cavernous sinus and returning via the jugular vein to the heart (Johnsen *et al*., 1985, 1987; Johnsen and Folkow, 1988). Although this differential vasoconstriction can modulate the degree of selective brain cooling, it does not fully explain the control of selective brain cooling in artiodactyls. In addition to the angularis oculi veins, less superficial veins, such as the sphenopalatine, external ophthalmic and ethmoidal veins, also supply venous blood to the cavernous sinus (Sisson and Grossman, 1967; Carlton and McKean, 1977). As a result, severing the angularis oculi veins does not completely eliminate selective brain cooling (Fuller *et al*., 2011). High sympathetic tone attenuates selective brain cooling not only by constricting the angularis oculi veins, but also by constricting nasal mucosal blood vessels and closing arteriovenous anastomoses. Within the nasal mucosa, the rate of heat extraction is attenuated through a combination of reduced blood flow and the restriction of airway width (Malm, 1973). Thus, increased cranial sympathetic nervous system activity decreases blood flow to the evaporating surfaces of the head as well as redirecting the flow of venous blood leaving those surfaces away from the cavernous sinus (Maloney and Mitchell, 1997; Fuller *et al*., 2011). These changes result in an upward shift of the threshold temperature for selective brain cooling, as documented in springbok (*Antidorcas marsupialis*; Mitchell *et al*., 1997), or complete abolishment of selective brain cooling, as documented in black wildebeest (*Connochaetes gnu*; Jessen *et al*., 1994). Free-living mammals rarely engage in intensive exercise except for predator–prey interactions, during which there is a dramatic increase in sympathetic nervous system activity in both predator and prey. The sympathetic activity prevalent during flight and fright in wild artiodactyls abolishes selective brain cooling (Jessen *et al*., 1994), overriding the drive of high body temperature. Selective brain cooling observed in tame artiodactyls exercising at low or moderate intensity is likely to be associated with low sympathetic activity (Mitchell *et al*., 2002).

Although it is not as strong as in intense exercise during predator–prey interactions, there is also sympathetic activation during psychological stress. The effects of psychological stress, including an increase in body and brain temperatures, are well documented, even in mammals lacking a carotid rete, such as rats (Mohammed *et al*., 2014). In mammals with a carotid rete, selective brain cooling is absent or reduced in other circumstances likely to be associated with increased sympathetic tone, including nearby human presence (Maloney *et al*., 2001), the return of drinking water to dehydrated artiodactyls (Fuller *et al*., 2007) and vigilance in male artiodactyls (Maloney *et al*., 2002; Hetem *et al*., 2012). The temperament of individual mammals, a heritable trait among artiodactyls (Murphy *et al*., 1994), plays an important role in how situations are experienced (Beausoleil *et al*., 2008). For example, individual roe deer (*Capreolus capreolus*) manifest different responses to stress (Monestier *et al*., 2016) and therefore, presumably, different levels of sympathetic activation. Individual variability in sympathetic responses to the same stressor may contribute to an underlying plasticity in the control of selective brain cooling (Strauss *et al*., 2016).

#### Hydration status

**Figure 4:**
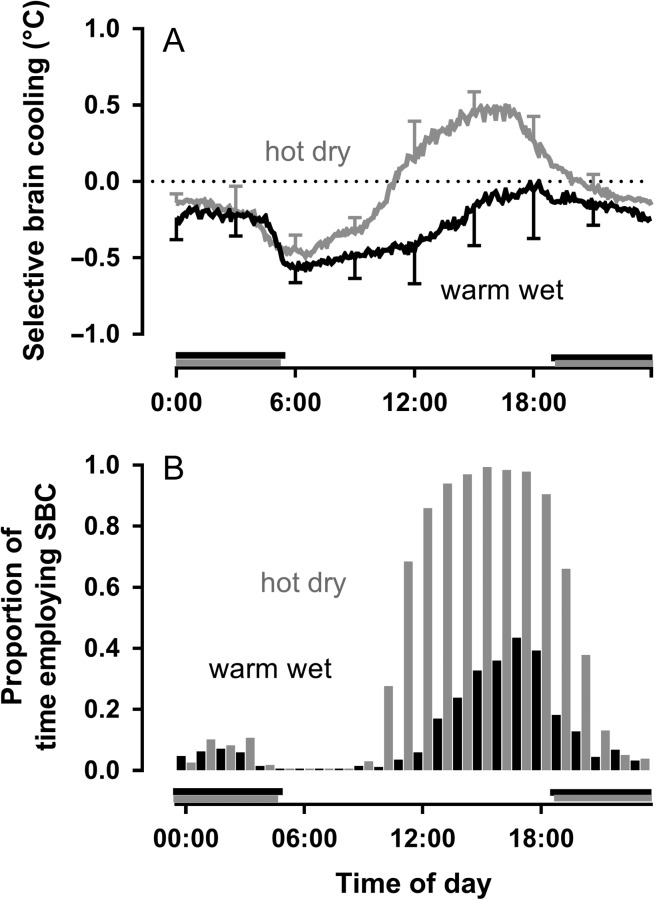
The effect of aridity on selective brain cooling as illustrated through differences in the mean (±SD) magnitude of selective brain cooling (carotid blood temperature minus hypothalamic temperature; **A**) and the proportion of time that a single Arabian oryx used selective brain cooling in the hot, hyper-arid deserts of Saudi Arabia (**B**). Grey depicts the hot dry and black the warm wet periods. Horizontal grey and black bars depict night-time during the two periods of interest. Data from Hetem *et al*. (2012).

**Figure 5:**
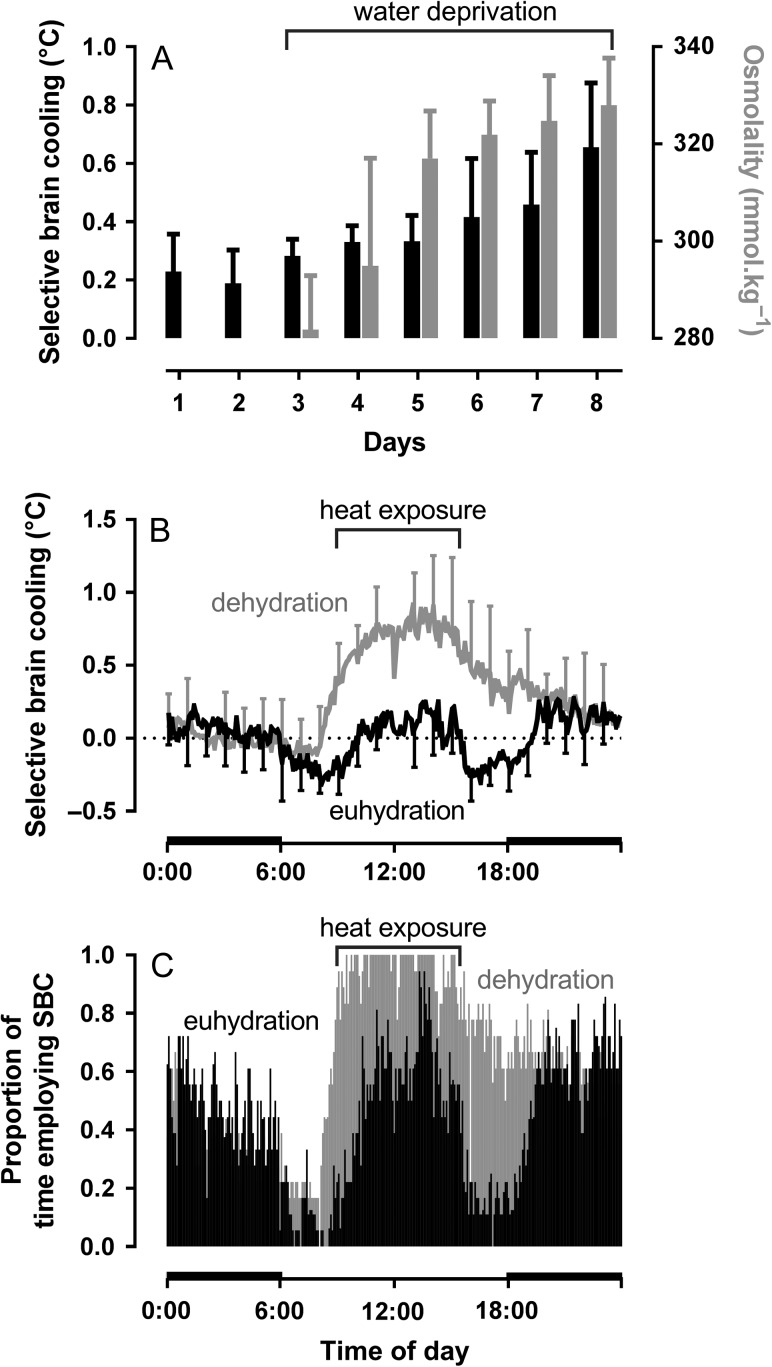
Summary of the effects of water deprivation on selective brain cooling in Dorper sheep. (**A**) The relationship between the mean (±SD) magnitude of selective brain cooling (carotid blood temperature minus hypothalamic temperature, black bars) and osmolality (grey bars) during 5 days of water deprivation; the black bracket indicates the period of water deprivation. (**B**) The mean (±SD) 24 h magnitude of selective brain cooling during euhydration (black line, days 1 and 2 in A) and dehydration (grey line, days 7 and 8 in A); horizontal black bars along the *x*-axis depict night-time. (**C**) The proportion of time that the sheep spent using selective brain cooling throughout the 24 h period when euhydrated (black bars; days 1 and 2 in A) and when dehydrated (grey bars; days 7 and 8 in A); the black bracket indicates the period of heat exposure (09:00–15:00) and the horizontal black bars along the *x*-axis depict night-time. Data from Strauss *et al*. (2015).

Selective brain cooling is enhanced during water deprivation (Jessen *et al*., 1998; Fuller *et al*., 2007; Strauss *et al*., 2015). Given that body temperature is elevated in dehydrated mammals exposed to heat, that enhancement could arise from a stronger thermal drive on selective brain cooling (Jessen *et al*., 1998). However, Fuller *et al*. (2007) showed that sheep exhibited a higher magnitude of selective brain cooling during dehydration even when carotid arterial blood temperature did not increase. Water deprivation on its own therefore seems to be a sufficient stimulus to enhance selective brain cooling. That idea is supported by measurement of selective brain cooling in antelope living free in arid environments. In the hyper-arid desert of Saudi Arabia, the mean magnitude of selective brain cooling in free-living Arabian oryx (*Oryx leucoryx*) peaked in the afternoon (Fig. 4A), well after solar noon and maximal heat load, as measured with miniature black globe thermometers on the collar of the animals (Hetem *et al*., 2007). Water availability, or aridity, appeared to have been the main factor determining the use and magnitude of selective brain cooling, with selective brain cooling being enhanced in the dry period compared with the wet period (Hetem *et al*., 2012). During the hot dry period when no drinking water was available, the Arabian oryx made near-continuous use of selective brain cooling during the 4 h leading to sunset (Fig. 5B, grey bars), compared with no more than 40% of the time during the warm wet period, when the oryx presumably had access to drinking water or food with a higher moisture content (Fig. 4B, black bars). Despite being exposed to similar ambient temperatures, Arabian oryx, in the hyper-arid desert of Saudi Arabia, also showed enhanced selective brain cooling compared with the congeneric gemsbok with free access to water (Maloney *et al*., 2002), as they initiated selective brain cooling at a lower threshold temperature and used selective brain cooling more frequently and at greater magnitude than did the gemsbok (Hetem *et al*., 2012).

The enhancement of selective brain cooling by water deprivation has been confirmed in more controlled conditions, in which domestic artiodactyls can be handled for sample collection (Jessen *et al*., 1998; Fuller *et al*., 2007; Strauss *et al*., 2015). When Dorper sheep (*Ovis aries*) were deprived of drinking water and exposed to diurnal heat load, after the third day of water deprivation, when the water reservoir within the rumen would have been depleted, plasma osmolality and selective brain cooling magnitude increased in a near-linear manner and in unison (Fig. 5A; Strauss *et al*., 2015). After 5 days of water deprivation, the 24 h mean magnitude of selective brain cooling was three times greater (0.9 vs. 0.3°C) than that during euhydration (Fig. 5B). The proportion of time during which the sheep used selective brain cooling also differed markedly depending on hydration status. Figure 5C shows that the sheep used some selective brain cooling at all times of the day regardless of hydration status. However, when dehydrated, the sheep used selective brain cooling more frequently at all times of day, but especially during the daylight hours when selective brain cooling, on average, was used 75% of the time. Water deprivation, presumably acting through hyperosmolality, therefore appears to be a driver of selective brain cooling.

## Selective brain cooling conserves body water

In dehydrated mammals, increased osmotic pressure of the arterial blood perfusing the brain inhibits thermoregulatory responses to heat (McKinley *et al*., 2008), including evaporative heat loss (Doris, 1983). If hyperosmolality also enhances selective brain cooling, then what would be the benefit to artiodactyls? In goats, at least, but presumably also in other artiodactyls, the neural drive for respiratory evaporative heat loss is provided about equally by thermoreceptors in the hypothalamus and thermoreceptors within the trunk (Jessen and Feistkorn, 1984). During selective brain cooling, input from the hypothalamic temperature sensors would be attenuated. Consequently, panting and sweating, the main physiological avenues of evaporative heat loss in artiodactyls, would be attenuated. The potential water savings as a result of selective brain cooling could be substantial considering that a 1°C decrease in brain temperature resulted in a ~6-fold decrease in respiratory evaporative water loss in goats (Kuhnen and Jessen, 1994). The hypothalamic thermosensitivity for evaporative water loss in goats is therefore high. We calculated the hypothalamic thermosensitivity for evaporative water loss for goats, at a trunk temperature of 40°C, to be 0.35 W kg^−1^ °C^−1^, or 14 g H_2_O min^−1^ kg^−1^ °C^−1^ using data provided in Jessen and Feistkorn (1984). In contrast, for a species lacking a rete, the rabbit, the hypothalamic thermosensitivity for evaporative water loss, calculated at an ambient temperature of 39°C, was 0.038 W kg^−1^ °C^−1^, or 1.5 g H_2_O min^−1^ kg^−1^ °C^−1^ (Stitt, 1976). The hypothalamic thermosensitivity is therefore 10 times greater in the heat-exposed goat than in the heat-exposed rabbit. A relatively small change in the hypothalamic temperature of the goat (tenths of a degree Celsius) therefore can result in significant water savings as a result of decreased evaporative water loss.

In an elegant experiment on goats, Kuhnen (1997) used extracorporeal heat exchangers to manipulate selective brain cooling and measured respiratory evaporative heat loss. The experimental inhibition of selective brain cooling resulted in a reduced trunk threshold for respiratory evaporative heat loss; therefore, evaporative water loss occurred at lower body temperatures, as well as at a higher overall rate. At an aortic blood temperature of 40°C, selective brain cooling of only 0.5°C reduced respiratory water loss by 0.72 l day^−1^, the equivalent of 35% of the average daily water requirement of the goats (Kuhnen, 1997). Sweating also is driven largely by thermal receptors in the hypothalamus (Smiles *et al*., 1976); therefore, selective brain cooling also will reduce water loss by sweating (Strauss *et al*., 2015), further contributing to the water savings of a mammal that uses both forms of evaporative heat loss.

Recently, in the laboratory, we quantified the total water savings attributable to selective brain cooling in Dorper sheep. We used the stable hydrogen isotope deuterium oxide (D_2_O) to measure water turnover in sheep that were deprived of drinking water and naturally making use of selective brain cooling. The sheep lost a quarter of their body water over 5 days of water deprivation. The threshold temperature for selective brain cooling remained unchanged, but those individuals that used selective brain cooling more frequently, or of greater magnitude, had lower water turnover rates (they therefore conserved body water better) than did conspecifics that used selective brain cooling less frequently or of smaller magnitude. We showed that a 50 kg sheep that used selective brain cooling for half of a day would save 2.4 litres of water that day, the equivalent of ~60% of the daily water requirement of a Dorper sheep not exposed to heat (Strauss *et al*., 2015).

Thus, the reduction of hypothalamic temperature by the 1°C or less that selective brain cooling can achieve reduces evaporative cooling during heat exposure sufficiently to save a substantial portion of the water that an artiodactyl would need to access each day. How does an artiodactyl continue to maintain heat balance if its evaporative cooling is attenuated? The attenuation of evaporative cooling by selective brain cooling results in an increase in body temperature, including skin temperature (Caputa *et al*., 1986b; Laburn *et al*., 1988). In environments in which an artiodactyl can lose heat by radiation and convection, the higher skin temperature will enhance radiant and convective heat loss, so selective brain cooling will switch heat loss from evaporative to non-evaporative channels. If an artiodactyl is in an environment in which it is gaining heat, the rate of heat gain will be reduced by the higher skin temperature. In the process of conserving water, however, the artiodactyl may store heat during hot periods of the day. As selective brain cooling generally is used late in the afternoon (Fig. 2, left panels), the heat that is gained as a result of the suppression of evaporative cooling may be dissipated non-evaporatively during the night.

## Selective brain cooling as a physiological feature for surviving hotter and drier environments

Water economy strategies in artiodactyls provide compelling evidence that selective brain cooling can mitigate negative population responses to warming and aridification. The use of selective brain cooling offers significant water savings to artiodactyls, but it is not the only mechanism by which they can save water. Dehydration itself reduces evaporative water loss via osmosensitive neurons in the hypothalamus, even in the absence of selective brain cooling (Baker and Doris, 1982). However, unlike dehydration or blood osmolality, selective brain cooling can be switched off rapidly (probably within seconds) by high cranial sympathetic activity. That property of selective brain cooling potentially conveys a survival benefit (Mitchell *et al*., 2002). For example, should an artiodactyl in a hot environment and implementing selective brain cooling be confronted by a flight-or-fight situation, its selective brain cooling would be abolished immediately by increased sympathetic tone. Consequently, hypothalamic temperature would increase, and the hypothalamic drive on evaporative cooling would be restored immediately, with the full power of evaporative cooling invoked to dissipate the extra metabolic heat. Immediate survival outweighs the longer-term benefits of body water conservation, and the artiodactyl makes temporary use of full evaporative cooling to avoid a potentially lethal hyperthermia. When the threat has receded, selective brain cooling can again be initiated and evaporative water loss suppressed. Thus, selective brain cooling may bestow benefits for survival in arid environments in two ways: (i) switching selective brain cooling on conserves body water, improving long-term survival; whereas (ii) switching selective brain cooling off rapidly accelerates evaporative cooling to avoid lethal hyperthermia, thereby supporting immediate survival.

**Figure 6:**
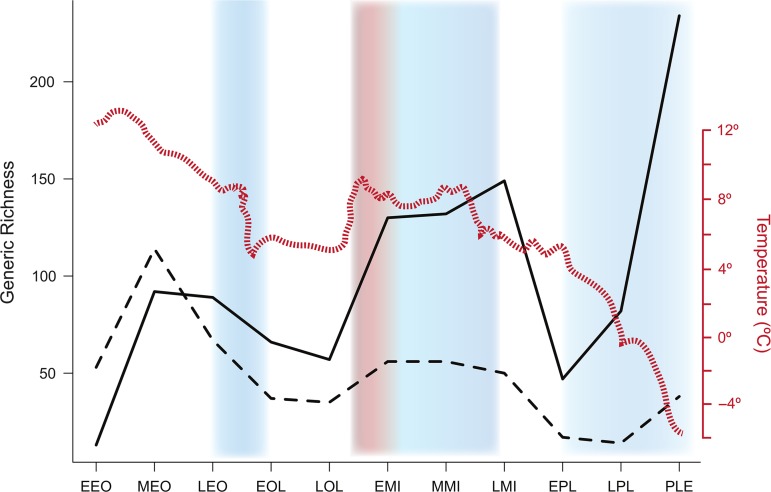
Diversity curve for the artiodactyls (solid line) and perissodactyls (dashed line) from the early Eocene (EEO) to the Palaeocene (PLE) relative to global temperature (red line; Zachos *et al*., 2001) and an indication of the relative hydrological regimes (Janis, 2008); blue and red shading represent aridification combined with cooling and warming temperatures, respectively. Occurrence data were downloaded from the Fossilworks/Paleobiology Database in August 2016. The epochs along the *x*-axis are as follows: EEO, Early Eocene; MEO, Mid-Eocene; LEO, Late Eocene; EOL, Early Oligocene; LOL, Late Oligocene; EMI, Early Miocene; MMI, Mid-Miocene; LMI, Late Miocene; EPL, Early Pleistocene; LPL, Late Pleistocene; and PLE, Paleocene.

The ability to modulate evaporative water loss through the use of selective brain cooling may have contributed to the evolutionary success of artiodactyls (Mitchell and Lust, 2008). It can be assumed that the carotid rete, and therefore selective brain cooling, evolved concomitantly with the emergence of the modern artiodactyl orders, at least 45 million years ago (Janis, 2009). When the diversity trends of Artiodactyla (presumably all rete bearing) and their sister clade Perissodactyla (presumably not rete bearing) are compared across the Cenozoic, discrepancies in generic richness are established in three pulses, each of which corresponds to a trend of aridification, whether warming or cooling (Fig. 6). In the earlier half of the Cenozoic, global climate is best described as a ‘tropical hothouse’ (Buchardt, 1978; Wolfe, 1978; Wing, 1987; Huber and Sloan, 2001; Bowen and Zachos, 2010; Galeotti *et al*., 2010). During this period of abundant moisture, artiodactyls and perissodactyls enjoyed similar generic richness (Fig. 6). However, a pronounced period of cooling and drying across the Eocene to Oligocene transition began to shift this relationship (Diester-Haass and Zahn, 1996; Lear *et al*., 2008). Although many large-bodied mammals faced extinction throughout this period (Prothero, 1985; Hooker, 1992; Legendre and Hartenberger, 1992), artiodactyls enjoyed a comparatively higher degree of survivorship both during and after this event. Throughout the drier, temperate Oligocene (Kennett, 1985; Ehrmann and Mackensen, 1992), artiodactyl generic richness remained elevated relative to perissodactyls (Fig. 6). Across the Late Oligocene to Early Miocene transition, another period of aridification swept the globe, this time accompanied by warming (Kennett, 1985; Retallack, 2013). This period of warming and aridification saw a pulse in generic richness of artiodactyls capable of selective brain cooling, immediately before the Mid-Miocene expansive radiation of C_4_ grasslands. It is often the expansion of grasslands and the artiodactyl rumen that have been associated and promoted as the driver of artiodactyl success (Retallack, 1991, 2001, 2007a, b, 2013; Jacobs *et al*., 1999). Although the late Cenozoic (Mid-Miocene to present) diversification patterns are largely attributable to the ruminant digestive physiology of bovids (Janis *et al*., 1998; Clauss *et al*., 2003; Janis, 2007, 2009; Clauss and Rössner, 2014; O'Brien, 2016), this highly specialized mode of digestion is variably present and cannot solely be responsible for earlier Cenozoic survivorship and diversification patterns. Indeed, it is likely that the influence of selective brain cooling insulated artiodactyls from extensive periods of aridification, whether warming or cooling, that saw declines among non-selective-brain-cooling ungulates (O'Brien, 2016).

This pattern of selective-brain-cooling-related survivorship is corroborated by evolutionary patterns of the Tragulidae. Although they are small-bodied artiodactyls with a rudimentary capacity for rumination, tragulids do not possess a carotid rete. Therefore, they can be considered to ruminate but not to selective brain cool. The earliest record of tragulids and other closely related small-bodied primitive ruminants is the Late Eocene to Early Oligocene (Webb and Taylor, 1980), when swamps and temperate forests dominated much of Europe and North America (Mai, 1989; Pross *et al*., 2001; Retallack, 2007a, b, 2013; Kunzmann, 2012). Since that period, these non-selective-brain-cooling taxa have experienced declines in generic richness even as more advanced ruminants have increased in diversity (Clauss and Rössner, 2014). Thus, although diet undoubtedly plays a role in the extinction and survivorship capacity of any taxon, failure to incorporate selective brain cooling not only leaves early artiodactyl evolution unsatisfactorily explained, but it renders an incomplete evaluation of these species’ abilities to persist into the Anthropocene.

Among artiodactyl species, there may be innate differences between species in their capacity for selective brain cooling, which may facilitate the persistence of those species with enhanced selective brain cooling in a hotter and drier Anthropocene. Although it has not been possible logistically, to date, to measure the water savings that result from selective brain cooling in free-living artiodactyls, we recently investigated selective brain cooling in three sympatric antelope species with different water dependencies and determined the dimensions of their carotid retes (Strauss *et al*., 2016). Individuals of all three species, living free in their natural habitats under the same environmental conditions, used selective brain cooling, but we found no differences in selective brain cooling use between the gemsbok that is independent of surface water, the red hartebeest of intermediate water dependency and the blue wildebeest that is dependent on surface water and has to drink water daily. In fact, we found more variability in selective brain cooling use within those species than between those species in a habitat in which surface water was available *ad libitum*. Although earlier descriptions of carotid rete morphology documented variability in the vascularization of the rete between species (Daniel *et al*., 1953; Carlton and McKean, 1977), we found little quantifiable difference in the rete anatomy of those three artiodactyls with varying ecological water requirements (Strauss *et al*., 2016). The observed variability in use of selective brain cooling within these three species living in exactly the same environmental conditions reaffirms the concept that selective brain cooling is not simply under thermal control (in the sense that there is a threshold temperature at which it is initiated). Moreover, the plasticity in the use of selective brain cooling may provide a physiological feature for selection in the face of anthropogenic climate change, as numerous regions are expected to become hotter and drier, with increased variability in rainfall (Niang *et al*., 2014). Large mammals will be unable to adapt genetically given the rapid rate of climate change and will be unlikely to be able to move to new, suitable habitats, leaving them dependent on phenotypic plasticity if they are to counter such climate change (Hetem *et al*., 2014).

Having survived and diversified dramatically during periods of pronounced aridification, the artiodactyls may be insulated, to a degree, from global warming and drying (Mitchell and Lust, 2008). Moreover, the observed inter-individual variability in selective brain cooling use within species implies that individual artiodactyls within populations, regardless of species or ecological water dependency, might have a relative evolutionary advantage in hotter, drier, less predictable environments. Representatives of the characteristic large herds of artiodactyls across various landscapes, which often also form the mainstay of large tracts of land under conservation management, therefore could persist through the Anthropocene. However, considering the continuing decrease in antelope populations across the globe (Ripple *et al*., 2015) and the poor performance of artiodactyls during mid-latitude glaciation of the Miocene to Pliocene transition, persistence through the Anthropocene may depend on the degree of intra-specific selective brain cooling plasticity. Indeed, artiodactyls such as the Arabian oryx may already be living close to their physiological tolerance levels. With a 24 h body temperature amplitude (maximum minus minimum 24 h body temperature) of up to 7.7°C during the summer, these animals are seasonally losing control of homeothermy as a result of water and nutritional stress (Hetem *et al*., 2012, 2016). As a consequence of a paucity of similar data from other artiodactyls, and also from perissodactyls, we know little about the physiological performance of these mammals under current environmental conditions and, ultimately, their ability to cope with a changing climate. Because perissodactyls, many of which are evolutionarily distinct (Zoological Society of London, 2014), cannot use selective brain cooling to conserve body water, concerted conservation efforts may be required as conditions become drier under anthropogenic climate change. Yet such conservation efforts should not be to the detriment of other species sensitive to disturbance (see, for example, Harrington *et al*., 1999).

Survival of those predators depredating artiodactyls and perissodactyls may also depend on the predators’ capacity for selective brain cooling, as selective brain cooling has been observed in heat-stressed domestic cats (Baker and Doris, 1982) and dogs (Baker, 1984). The degree to which free-living felids and canids use selective brain cooling is currently not known. We also do not know, therefore, whether the evolutionary success of carnivores can be attributed to the development of their carotid rete, selective brain cooling and the conservation of body water. A better understanding of selective brain cooling, as a form of physiological plasticity that is available to some mammals, is integral to efforts to predict how mammals will respond to changing environments and how best to conserve them. The studies that are required are long-term investigations undertaken on a range of artiodactyl and carnivore species (Fuller *et al*., 2014; Hetem *et al*., 2014). Such studies should focus on identifiable individuals and their progeny, free-living in their natural environment, with the aim of relating varying levels of evolutionary success to the observed flexibility in selective brain cooling and resulting water savings.
